# Randomized controlled trial of liberal vs. standard fasting instructions in percutaneous cardiac procedures

**DOI:** 10.1186/s13741-023-00333-z

**Published:** 2023-08-08

**Authors:** Derek J. Atkinson, Jamie L. Romeiser, Ibrahim O. Almasry, Henry J. Tannous, Puja B. Parikh, Elliott Bennett-Guerrero

**Affiliations:** 1 Departments of Anesthesiology (DA, JLR, EBG), Medicine/Cardiology (IA, PP), and Surgery/Cardiothoracic (HT), Stony Brook University Medical Center, 101 Nicolls Road, Stony Brook, NY 11794 USA; 2 Department of Public Health and Preventive Medicine, SUNY Upstate Medical University, Syracuse, NY USA

## Abstract

**Background:**

Pre-procedural fasting to reduce aspiration risk is usual care prior to surgery requiring anesthesia. Prolonged fasting, however, can result in dehydration and may adversely affect patient experience and outcomes. Previous studies suggest that providing a supplemental beverage to patients undergoing cardiac and a variety of other surgical procedures improves patients’ subjective assessment of thirst and hunger and potentially decreases the need for inotrope and vasopressor therapy. Less is known, however, about the effects of ad libitum clear liquids up to 2 h prior to surgery.

**Methods:**

Adult patients undergoing transcatheter aortic valve replacement (TAVR) or arrhythmia ablation were randomized (1:1) to ad libitum clear liquids up to 2 h prior to their procedure vs. *nil per os* (NPO) after midnight (control group, usual care). The primary endpoint was a composite satisfaction score that included patient-reported thirst, hunger, headache, nausea, lightheadedness, and anxiousness prior to surgery. The incidence of case-delay was recorded. Intraoperative vasopressor administration, changes in creatinine, anti-emetic use, and hospital length of stay (LOS) were recorded. Safety endpoints including aspiration were assessed.

**Results:**

A total of 200 patients were randomized and 181 patients were included in the final analysis. Overall, 92% of patients were ASA class III or IV and 23% of patients had NYHA class III or IV symptoms. Groups were well balanced with no significant differences in age, sex or baseline cardiac or renal disease. The composite satisfaction score (primary endpoint) was not significantly different between groups (*Ad libitum* median = 12, IQR = [6, 17], vs Standard NPO median = 10, IQR = [5, 15], [95% CI = [-1, 4]). No significant differences between the two groups were observed in any of the individual survey questions (thirst, hunger, headache, nausea, lightheadedness, anxiousness). No significant differences between groups were observed for intra-operative vasopressor use, changes in creatinine, rescue anti-emetic use or hospital LOS. There were no case delays attributed to the intervention. There were no cases of suspected aspiration.

**Conclusion:**

No adverse events or case delays were observed in the ad libitum clears group. No significant benefit, however, was observed in patient satisfaction or any of the pre-specified secondary endpoints in patients randomized to ad libitum clear liquids up to 2 h prior to their procedure.

**Trial registration:**

NCT04079543.

**Supplementary Information:**

The online version contains supplementary material available at 10.1186/s13741-023-00333-z.

## Background

The practice of pre-operative fasting has been a focal point of anesthesiologists’ practice since the 1940’s when Mendelson described the incidence of pulmonary aspiration in an obstetric population receiving anesthesia and suggested fasting as a means to reduce this serious complication (Mendelson 1946). More recently, however, there has been a recognition that there may be adverse effects from lengthy pre-procedural fasting, e.g. the potential for dehydration and its effects on subjective patient well-being and peri-procedural outcomes (Hausel et al. 2001; Tran et al. 2013; Breuer et al. 2006). Less restrictive protocols for fasting have been studied in a healthy population and deemed safe for this group (Brady et al. 2003). Reduced fasting times, often in combination with a carbohydrate beverage have been associated with improved patient comfort, (Cheng et al. 2021) decreased gastric volume, (Brady et al. 2003; Järvelä et al. 2008; Yildiz et al. 2013) decreased hypotension, (Breuer et al. 2006; Simpao et al. 2020; Şavluk et al. 2017), decreased cortisol (Gümüs et al. 2021) and interleukin 6 levels, (Rizvanović et al. 2019) decreased post-operative delirium, (Radtke et al. 2010) and reduced hospital length of stay (LOS) (Cheng et al. 2021; Smith et al. 2014; Awad et al. 2013). Based in part on these data, the American Society of Anesthesiologists (ASA) practice guidelines currently recommend allowance of clear liquids up to 2 h prior to anesthesia for *healthy* patients undergoing elective procedures (Practice Guidelines for Preoperative Fasting and the Use of Pharmacologic Agents to Reduce the Risk of Pulmonary Aspiration: Application to Healthy Patients Undergoing Elective Procedures: An Updated Report by the American Society of Anesthesiologists Task Force on Preoperative Fasting and the Use of Pharmacologic Agents to Reduce the Risk of Pulmonary Aspiration 2017).

There are, however, still several gaps in knowledge related to peri-operative fasting. First, as suggested by the ASA guidelines, much of prior work has focused on “healthy” patients, and many patients undergoing cardiac and other complex procedures do not fit into this category. Second, and perhaps more importantly, previous studies have mostly prescribed fluid, usually a carbohydrate beverage, to patients in the reduced fasting arms, rather than allowed patients to drink clear liquids as they wished, i.e. ad libitum. Our study sought to compare traditional, after midnight pre-procedural fasting instructions versus ad libitum*,* clear liquids up to 2 h instructions, prior to elective surgery in a “non-healthy” group of patients requiring general anesthesia or monitored anesthesia care. We hypothesized that allowing ad libitum clear liquids up to 2 h prior to the procedure improves patient satisfaction in this cohort.

## Methods

### Study design and patient population

This prospective, unblinded, randomized controlled trial was conducted following approval by the Stony Brook University Hospital Institutional Review Board (protocol IRB2019-00261). Registration of the trial (Clinicaltrials.gov identifier: NCT04079543) occurred prior to initiation of enrollment. Written informed consent was obtained from all participants.

Individuals over the age of 18 and scheduled for elective Transcatheter Aortic Valve Replacement (TAVR) or arrhythmia ablation were eligible to participate. At our hospital all patients undergoing these procedures receive either general anesthesia or monitored anesthesia care. At our institution, monitored anesthesia care for these procedures usually consists of administration of a short acting narcotic and/or benzodiazepine in combination with a dexmedetomidine or propofol infusion titrated to a Richmond Agitation Sedation Scale (RASS) of -3 to -4. Subjects were ineligible if they were unable to provide written informed consent or lacked the ability to answer the survey questions. Patients undergoing emergency procedures, those with a prior history of aspiration or dysphagia, those receiving nutrition via a feeding tube or total parenteral nutrition, and those receiving active treatment for gastroparesis with pro-motility agents were excluded.

### Interventions

Patients randomized to the traditional *nil per os* (NPO) group were instructed to fast after midnight prior to the procedure, except for a sip of water with scheduled medications. Patients in the ad libitum clears group were instructed that they could consume unlimited clear liquids, of their choosing, up to 2-h prior to the scheduled procedure start time. Patients received their instructions at the time of randomization at the pre-operative visit and received a follow up phone call which reinforced these instructions the day before the procedure. All other nursing, anesthesia and surgical care was routine throughout the pre, intra and post-operative periods.

### Endpoints and data collection

#### Primary endpoint

On the day of the procedure patients completed a patient satisfaction survey 2 h (± 2 h) prior to surgery (Additional file 1: Appendix A). Components of the satisfaction survey included patient self-reported thirst, hunger, headache, nausea, lightheadedness and anxiousness. Patients were asked to rate each component from 0–10 along a visual analog scale. A composite “patient satisfaction” score with a range of 0–60 was calculated from these six answers, with lower values indicating greater satisfaction.

#### Prespecified secondary endpoints

The administration of vasopressors was collected from the electronic anesthesia record. Vasopressor requirement was defined a priori as the administration for greater than or equal to 15 min during the procedure to maintain adequate arterial blood pressure as determined by the attending anesthesiologist of either phenylephrine ≥ 40 mcg/min, norepinephrine ≥ 4 mcg/min or any dose of vasopressin infusion. Post-operative nausea and vomiting was defined as receiving a dose of rescue anti-emetic (ondansetron, promethazine, or metoclopramide) within the first 24 h post-procedurally. Post-operative hospital length of stay was defined as the number of days from the day of procedure until discharge from the hospital. Change in serum creatinine was defined as the change from baseline pre-procedural creatinine to the highest creatinine value within the first 72 h post-procedurally. The incidence and cause of case cancellation and significant case delay, defined as having an actual case start time that was greater than 60 min past the scheduled start time, was collected.

#### Prespecified safety endpoints

Possible aspiration events were monitored throughout the study using the following methods. First, anesthesia records were examined for any notation of intra-operative regurgitation, vomiting or aspiration. Second, the EMR was reviewed for evidence of a new or increased supplemental oxygen requirement at 24 h ± 2 h post-procedurally. Third, any chest radiographs taken within the first 72 h post-procedurally were analyzed for signs of probable or definite aspiration as read by the attending radiologist.

### Data management and statistical analysis

All data and statistical methods were overseen by the trial’s statistician (co-author JLR). Data were recorded into a dedicated REDCap software database for this trial only accessible by study personnel.

### Randomization

Randomization lists were generated by the trial’s statistician using block randomization with random permuted block sizes of 4, 6, and 8 to ensure a similar number of patients in each group over time. These lists were stratified by procedure type (TAVR and arrhythmia ablation), uploaded and implemented using REDCap software’s randomization feature.

### Sample size

Historical quality assurance data from a *Carbohydrate Beverage Satisfaction Survey* at our institution were used to estimate the sample size needed to detect a significant difference between the two groups. These surveys contained the same questions as our primary endpoint satisfaction survey, and were conducted in two cohorts that were under similar conditions as the ones specified in this trial. Based on these data, we estimated and powered the study to detect a 5 point difference in satisfaction scores (maximum difference of 60 points) between the two groups, with a 10 point standard deviation. Using an alpha of 0.05, with power = 0.85, the estimated number of subjects needed for each group was at least 73, for a total of at least 146 subjects. To increase confidence in exploring our secondary outcomes, we allowed for enrollment of a total of 200 patients, assuming that with screen failures the study would remain adequately powered.

### Statistical tests

Categorical variables are described as frequencies and percentages, and continuous variables are described as either means (standard deviation [SD]) or medians (interquartile range [IQR]). Relative differences between groups (ad libitum minus standard) are reported for variables described using percentages and means, including a 95% confidence interval for the difference in primary and secondary outcomes. Median differences between groups were calculated using Hodges-Lehmann estimation location shifts, including a 95% confidence interval for primary and secondary outcomes. Composite satisfaction scores, change in creatinine, and postoperative LOS were tested for normality using Shapiro–Wilk tests, and compared by treatment group using Wilcoxon Rank Sum Tests. Use of vasopressors, incidence of any post-operative nausea and vomiting, intra-operative vomiting, and increased oxygen use at 24 h were assessed using chi-square tests and fisher’s exact tests. All analyses were performed using SAS 9.4 © software (Cary, NC).

## Results

**Fig. 1:**
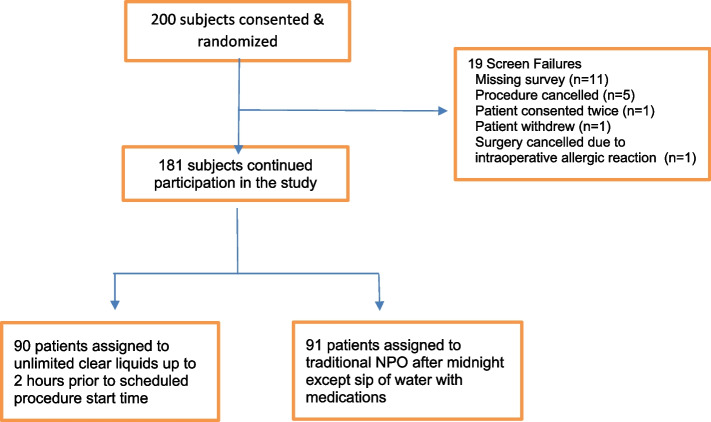
CONSORT Diagram

A total of 200 patients were consented between September 24, 2019 and July 27, 2021. Of these, 19 patients were excluded, which resulted in 181 subjects analyzed (Fig. 1- CONSORT diagram with reasons for exclusion).

Patient characteristic were similar between the ad libitum reduced fasting and standard NPO groups (Table 1: Demographics). Patients were similar in age, sex, race and co-morbidities including ASA score, LVEF and NYHA class. Ninety-two percent of patients were ASA class III or IV and 23% of patients had NYHA class III or IV symptoms. More patients had a history of post-operative nausea and vomiting (PONV) in the ad libitum group compared to the standard group (difference—6.7%), but this group had a smaller percentage of patients with obstructive sleep apnea (OSA) (difference -15.2%).

**Table 1:** Demographics and comorbidities

	**Ad libitum clears**		**Usual Care**		**Difference**
Patients in each study arm	90	49.7%	91	50.3%	-0.55%
Planned Procedure—n (%)					
Transcatheter Aortic Valve Replacement	21	23.3%	24	26.4%	-3.0%
Arrhythmia Ablation	69	76.7%	67	73.6%	3.0%
Sex (Male)- n (%)	58	64.4%	63	69.2%	-4.8%
Age—mean (SD)	68.1	11.8	68.9	11.5	-0.8
Race- n (%)					
Asian	1	1.1%	1	1.1%	0.0%
Black/African American	4	4.4%	2	2.2%	2.2%
White	85	94.4%	87	95.6%	-1.2%
Other	0	0.0%	1	1.1%	-1.1%
Smoking Status- n (%)					
Current	11	12.2%	6	6.6%	5.6%
Former	46	51.1%	44	48.4%	2.8%
Never	33	36.7%	41	45.1%	-8.4%
Hypertension—n (%)	66	73.3%	68	74.7%	-1.4%
Previous PONV- n (%)	7	7.8%	1	1.1%	6.7%^a^
Chronic Obstructive Lung Disease- n (%)	7	7.8%	8	8.8%	-1.0%
Home Oxygen- n (%)	2	2.2%	0	0.0%	2.2%
Coronary Artery Disease- n (%)	26	28.9%	21	23.1%	5.8%
Stent	20	22.2%	14	15.4%	6.8%
CABG	9	10%	9	9.9%	0.1%
Medically managed	1	1.1%	2	2.2%	-1.1%
Unknown	1	1.1%	1	1.1%	0.0%
Diabetes- n (%)	21	23.3%	20	22%	1.4%
Diet	3	3.3%	2	2.2%	1.1%
Insulin	5	5.6%	4	4.4%	1.2%
Oral	12	13.3%	14	15.4%	-2.1%
Unknown	1	1.1%	0	0.0%	1.1%
Congestive Heart Failure Class- n (%)	34	37.8%	47	51.6%	-13.9%
I	4	4.4%	7	7.7%	-3.2%
II	11	12.2%	16	17.6%	-5.4%
III	17	18.9%	22	24.2%	-5.3%
IV	2	2.2%	1	1.1%	1.1%
Unknown	0	0.0%	1	1.1%	-1.1%
Chronic Kidney Disease Class- n (%)	16	17.8%	15	16.5%	1.3%
III	4	4.4%	7	7.7%	-3.2%
IIIB	8	8.9%	3	3.3%	5.6%
IV	2	2.2%	3	3.3%	-1.1%
V	1	1.1%	2	2.2%	-1.1%
Unknown	1	1.1%	0	0.0%	1.1%
Obstructive Sleep Apnea- n (%)	13	14.4%	27	29.7%	-15.2%^a^
CPAP	8	8.9%	13	14.3%	-5.4%
GERD/Reflux- n (%)	23	25.6%	25	27.5%	-1.9%
No treatment	7	7.8%	3	3.3%	4.5%
Proton Pump Inhibitor	13	14.4%	19	20.9%	-6.4%
Histamine 2 Blocker	1	1.1%	3	3.3%	-2.2%
Unknown	2	2.2%	0	0.0%	2.2%
Glucose—median (IQR)	99	(88, 112)	103	(90, 166)	-2
Blood Urea Nitrogen—median (IQR)	19	(15, 25)	19	(15, 23)	0
Creatinine—median (IQR)	1	(0.8, 1.2)	1	(0.8, 1.2)	-0.1
Glomerular Filtration Rate—mean (std)	73.5	22.7	70.1	21.1	3.4
Hemoglobin—mean (std)	13.7	1.7	13.7	2	0.02
Complete ECHO Report in last 6 mo- n (%)	62	68.9%	62	68.1%	0
LVEF—median (IQR)	60.5	(50, 65)	60	(50, 65)	1
LVEF Category—n (%)					
Normal	44	48.9%	42	46.2%	2.7%
Mildly reduced	10	11.1%	13	14.3%	-3.2%
Moderately Reduced	3	3.3%	4	4.4%	-1.1%
Severely reduced	5	5.6%	2	2.2%	3.4%
Unknown	0	0.0%	1	1.1%	-1.1%

Number of patients with characteristic is followed by percentage or mean (std) or median (IQR) as appropriate

*LVEF* Left ventricular ejection fraction, *GERD* Gastro-esophageal reflux disease, *CABG* Coronary artery bypass grafting, *CPAP* Continuous positive airway pressure

^a^Indicates a significant difference between groups (*p* < 0.05). Median differences are calculated using the Hodges-Lehmann Estimation location shift. Frequency differences and mean differences are calculated as relative differences

Study groups were similar in all pre-operative and intra-operative variables (Table 2: Pre, Intra, and Post Op). There were 4 first case delays, all within with the standard NPO group. Patients in the ad libitum group had slightly higher but clinically insignificant post-procedural O_2_ saturation.

**Table 2:** Perioperative characteristics

**Pre-Op Intake**	**Ad libitum clears**		**Usual Care**		**Difference**
Height—mean (std)	173.1	11.3	174.8	11.4	-1.7
Weight—mean (std)	92	25.2	91.9	18.7	0.1
Heart rate—median (IQR)	69.5	(60, 83)	67	(60, 82)	2
Blood Pressure Systolic—mean (std)	132.3	19.8	137.4	21	-5
Blood Pressure Diastolic—mean (std)	74.8	12.1	75.1	10.8	-0.3
SpO_2_—median (IQR)	97	(96, 99)	97	(96, 98)	0
ASA Class—n (%)					
I	0	0.0%	0	0.0%	0.0%
II	5	5.6%	7	7.7%	-2.1%
III	57	63.3%	59	64.8%	-1.5%
IV	27	30%	25	27.5%	2.5%
Not available (surgery cancelled)	1	1.1%	0	0.0%	1.1%
Rhythm—n (%)					
Sinus Rhythm	48	53.3%	58	63.7%	-10.4%
Atrial Fibrillation	26	28.9%	23	25.3%	3.6%
Other/Not Documented	16	17.8%	10	11.0%	6.8%
Time from last clear liquid to Scheduled Procedure (minutes)—median (IQR)	210	(150, 510)	570	(210, 750)	-180^a^
First Case – n (%)	59	65.6%	58	63.7%	1.8%
First Case Delay (% of first cases) – n (%)	0	0.0%	4	6.9%	-6.9%
Duration of delay in minutes- median (IQR)	–	–	241	(159, 312)	–
**Intra-Op**	**Ad libitum clears**		**Usual Care**		**Difference**
Actual Procedure—n (%)					
Transcatheter Aortic Valve Replacement	21	23.3%	24	26.4%	-3.0%
Arrhythmia Ablation	68	75.6%	67	73.6%	1.9%
Other	1	1.1%	0	0.0%	1.1%
Type of Anesthesia—n (%)					
General with Endotracheal Tube	56	62.2%	57	62.6%	-0.4%
Monitored Aesthesia Care/Deep Sedation	33	36.7%	34	37.4%	-0.7%
Not applicable (surgery cancelled)	1	1.1%	0	0.0%	1.10%
Emesis—n (%)	0	0.0%	0	0.0%	0.0%
Total contrast recorded- n (%)	19	21.1%	26	28.6%	-7.5%
Total contrast—median (IQR)	60	(35, 70)	67.5	(40, 110)	-11
Vasopressor—n (%)	56	62.2%	55	60.4%	1.8%
Phenylephrine	49	54.4%	46	50.5%	3.9%
Epinephrine	0	0.0%	1	1.1%	-1.1%
Norepinephrine	7	7.8%	13	14.3%	-6.5%
Total Crystalloids—median (IQR)	1453	(700, 2000)	1375	(625, 2000)	40
Any Colloids—n (%)	1	1.1%	1	1.1%	0%
Estimated Blood Loss- median (IQR)	0	(0, 5)	0	(0, 20)	0
Any allogeneic red blood cells—n (%)	1	1.1%	1	1.1%	0%
Urine Output- median (IQR)	370	(0, 600)	225	(0, 510)	25
**Post-op**	**Ad libitum clears**		**Usual Care**		**Difference**
Heart rate—median (IQR)	65	(58, 74)	64	(56, 75)	0
Blood Pressure Systolic—mean (std)	118.2	21.3	123.1	22.4	-4.9
Blood Pressure Diastolic—mean (std)	63.3	12.1	64.7	12.6	-1.4
SpO_2_—median (IQR)	98	(96, 100)	97	(95, 99)	1^a^
Rhythm—n (%)					
Not Documented	10	11.1%	9	9.9%	1.2%
Sinus Rhythm	64	71.1%	65	71.4%	-0.3%
Atrial Fibrillation	4	4.4%	3	3.3%	1.1%
Other/Not Documented	22	24.4%	23	25.3%	-0.8%
Post-Op Labs Recorded—n (%)	61	67.8%	61	67%	0.7%
Glucose—median (IQR)	114.5	(104, 140)	118	(105, 132)	0
Bun—median (IQR)	18	(15, 21)	17.5	(14, 21)	0
Creatinine—median (IQR)	1	(0.7, 1.1)	1	(0.7, 1.1)	0.01
Supplemental O2—n (%)	8	8.9%	7	7.7%	1.2%
Ventilated postoperatively—n (%)	1	1.1%	0	0.0%	1.1%
Post-Op Opioids—n (%)	28	31.1%	24	-26.4%	4.7%
SpO2 = oxygen saturation					

Number of patients with characteristic is followed by percentage or mean (std) or median (IQR) as appropriate

^a^Indicates a significant difference between groups (*p* < 0.05). Median differences are calculated using the Hodges-Lehmann Estimation location shift. Frequency differences and mean differences are calculated as relative differences

Patient self-reported satisfaction scores (Table 3: Outcomes) were similar in both groups (Reduced fasting median = 12, IQR = [6, 17], vs Standard median = 10, IQR = [5, 15], [95% CI = [-1, 4]), and this small difference was not statistically significant (*p* = 0.18). In addition, no significant differences between study arms were observed for any of the 6 individual survey questions (thirst, hunger, headache, nausea, lightheadedness, anxiousness). Postoperative creatinine values, which were collected as part of routine clinical care, were available in the EMR for 117 patients. Changes in creatinine (mg/dL) were small in both groups (Ad libitum median = -0.08, IQR = [-0.15, 0.02], vs Standard median = -0.10, IQR = [-0.2,-0.02], difference -0.03, [95% CI = [-0.08, 0.02], *p* = 0.27. Post-operative length of stay, use of vasopressors, post-operative PONV, and all safety endpoints were similar between groups (Table 3).

**Table 3:** Primary and secondary endpoints

Outcomes	Ad libitum clears		Usual Care		*P*-Value	Difference	95% CI for difference
Patient Satisfaction Score—median (IQR)	12	(6, 17)	10	(5, 15)	0.18	2	(-1, 4)
Thirst	5	(2, 7)	4	(2, 5)	0.17	0	(0, 2)
Hunger	2.5	(0, 6)	2	(0, 5)	0.87	0	(0, 0)
Headache	0	(0, 0)	0	(0, 0)	0.46	0	(0, 0)
Nausea	0	(0, 0)	0	(0, 0)	0.32	0	(0, 0)
Lightheaded	0	(0, 0)	0	(0, 0)	0.84	0	(0, 0)
Anxious	3.5	(0, 7)	3	(0, 5)	0.35	0	(0, 1)
Change in Creatinine – n (%)	59	65.6%	58	63.7%	0.8	1.9%	(-12.1%, 15.8%)
Change in Creatinine (POST–PRE)—median (IQR)	-0.1	(-0.2, 0.02)	-0.1	(-0.2, -0.02)	0.27	-0.03	(-0.1, 0.02)
Vasopressor—n (%)	56	62.2%	55	60.4%	0.81	1.8%	(-12.4%, 16.0%)
PONV (rescue medication)	4	4.4%	4	4.4%	1	0.05%	(-5.9%, 6.0%)
PLOS [HRS]—median (IQR)	21.3	(7.9, 25.8)	21.3	(6.2, 25.2)	0.5	0.6	(-1.1, 2.6)
*Safety Endpoints*							
Intra-op Vomiting/ Regurgitation—n(%)	0	0%	0	0%	N/A	N/A	N/A
Post Op O_2_ needed at 24 h) – n (%)	8	8.9%	7	7.7%	0.77	1.2%	(-6.4%, 9.2%)
Evidence of Aspiration—n(%)	0	0%	0	0%	N/A	N/A	N/A

Number of patients with characteristic is followed by percentage or mean (std) or median (IQR) as appropriate

*PONV* Postoperative nausea or vomiting, *PLOS* Postoperative length of stay

## Discussion

In this randomized clinical trial, we observed no significant benefit to instructions of ad libitum clear liquids up to 2 h versus instructions of standard NPO after midnight in a group of “non-healthy” patients undergoing TAVR or arrhythmia ablation. Previous studies with a shortened fasting protocol, including a large meta-analysis of 5,606 patients (Cheng et al. 2021) demonstrated a significant benefit in subjective assessments of pre-operative well-being, including thirst, mouth dryness and hunger. Of note, most prior studies focused on a healthy patient population, which is consistent with the current ASA fasting guidelines deeming a more liberal protocol safe in “healthy” patients. In addition, the majority of previous studies used a protocol in which beverages, e.g. carbohydrate beverage, were prescribed to patients and included a defined intake volume and composition.

Our study differs from previous studies in several ways. First, patients in our study were allowed more flexibility in the volume and type of beverage consumed. This more pragmatic approach has the benefit of being simple to operationalize and thus potentially translatable to more centers/patients. Second, patients had a significant amount of cardiac dysfunction; 44.8% were previously diagnosed with congestive heart failure, 21% had documented reduced left ventricular ejection fraction on echocardiogram and 26% had known coronary artery disease. It is clinically accepted that patients with significant cardiac co-morbidities have narrower ranges of optimal intravascular volume and it has been posited that an ad libitum protocol might be more flexible and potentially safer for individual patients. Of note, it is unclear to what extent cardiac disease is in and of itself a risk factor for delayed gastric emptying. This is likely to be dependent on the severity of other comorbidities such as diabetes as well as the type and severity of cardiac dysfunction. In heart failure, for example, it has been postulated that several mechanisms may contribute to early satiety, delayed gastric emptying and nausea. These include an imbalance of sympathetic/parasympathetic tone, gut hypoperfusion and bacterial translocation leading to inflammation and volume overload leading to gut edema (Sundaram and Fang 2016). We are aware of only one study that attempted to measure gastric fluid volume in a cardiac surgical population. In this study by Bruer et al. patients undergoing CABG or valve surgery had gastric fluid volumes measured which were approximately normal in each arm (water vs carbohydrate drink vs prolonged fasting) (Breuer et al. 2006).

A potential downside, which may have contributed to our negative results, is that, in practice, many patients might not avail themselves of the opportunity to drink unlimited fluids up to 2 h before surgery due to a variety of factors. Other authors have pointed to examples of large differences in *actual* fasting times compared to provided fasting instructions, which are potentially explained by poor instructions, unintentional patient non-compliance or other undefined variables (Witt et al. 2021). While there was a significant difference in reported fasting times in our study, i.e. longer fasting time in the control group, the total volume was not recorded as we did not feel this was feasible to accurately record outside of the hospital. However, this may be an important variable. For example, Cheng et al. (Cheng et al. 2021) noted a trend in the literature for better mouth dryness, thirst and hunger scores with an increasing volume of prescribed liquid. Furthermore, the quality of the beverage may be important. In a modular update for 2023 the ASA modified its pre-operative fasting recommendations to specifically encourage consumption of a carbohydrate containing beverage 2 h prior to a procedure. This was based on superiority of the carbohydrate containing beverage versus non-carbohydrate clear liquid or traditional fasting, particularly as it related to pre-operative hunger (Joshi et al. 2023).

While our approach sought to be pragmatic for real world application, a limitation exists in determining the effect of compliance with instructions versus actual fluid intake. Anecdotal experience during our study suggested that some patients did not wish to drink clear liquids up to two hours prior to surgery because of previous instructions to fast after midnight for past surgeries. It is notable that a large percentage, i.e. 64%, of scheduled start times were “first cases”. At our institution first cases begin at 07:30 am, therefore patients would last be able to drink at around 5 am. This coincides with usual sleeping hours for most patients and is a relatively short fasting time, therefore, many patients in the ad libitum up to 2 h before surgery group might not have been thirsty this early in the morning. Nevertheless, an exploratory subgroup analysis of our non-first case patients did not show any difference, though this subgroup analysis is significantly underpowered. Finally, although not formally assessed in our study, many elderly patients have issues with incontinence. Thus, many patients in the more liberal NPO arm of our study may have avoided drinking fluids out of fear of not having easy access to bathroom facilities during their drive to the hospital or while waiting for their surgery. All of the above factors are potential reasons that patients who were randomized to ad libitum liquids up to 2 h prior to surgery may not have availed themselves of this opportunity. This may be important to investigators for future studies as well as to hospitals/clinicians who might consider a more liberal NPO instructions for some of their patients.

None of the secondary end-points were significantly different between the two fasting protocols. Several studies in cardiac surgery (Breuer et al. 2006; Şavluk et al. 2017; Feguri et al. 2019) have shown a trend towards decreased inotrope or vasopressor use in an oral carbohydrate loaded group. In contrast, studies on healthier patients having less invasive surgery have shown no difference (Asakura et al. 2015; Canbay 2017). In our study, a significant proportion of patients received vasopressors intra-operatively, however, there was no difference in this endpoint between the study groups. Potential explanations for this include the fact that patients received significant fluid intra-operatively, on average 1400 ml of crystalloid solution, which may have quickly restored intravascular volume. In addition, the studied procedures are considered minimally invasive as compared to open cardiac surgery and may present less of a surgical insult and concomitant stress response with its associated vasodilation and third spacing.

Comparable hemodynamics and adequate IV fluid administration, in addition to the low predicted incidence of AKI, makes the lack of difference between study arms in post-operative creatinine levels unsurprising. PONV was also similar between groups, which is consistent with previous studies and meta-analyses that did not find a correlation between fasting and pre-operative beverage intake (Cheng et al. 2021).

While our study showed no benefit to a more liberal NPO instruction, an important observation is that there did not appear to be any adverse events. We did not observe any suspected cases of aspiration, nor pre-operative volume overload or heart failure. Given the low incidence of aspiration overall, this study was underpowered to determine safety but may contribute to future analysis in answering this question in an older population with a high burden of co-morbidities. Importantly, we did not observe any case delays in patients randomized to the shorter NPO times. This is significant as case delays can have consequences on operating room efficiency and economics.

Limitations of our study include the unblinded study design and the subjective nature of the patient self-reported satisfaction survey. Also, the predefined vasopressor cut-off is a somewhat arbitrary number although this was modeled off of previous investigations (Tran et al. 2013; Şavluk et al. 2017). Strengths of the study include a randomized design with a relatively large sample size in a well-defined population.

In conclusion, we found that ad libitum clear liquids up to 2 h prior to surgery appeared to be safe with no adverse events or case delays associated with the intervention. No significant differences, however, were observed in our primary or any prespecified secondary endpoints between patients randomized to ad libitum clear liquids up to 2 h prior to TAVR or arrhythmia ablation versus traditional fasting. Based on our results, investigators wishing to study putative benefits of oral fluid loading prior to elective surgery, may consider prescribing, rather than allowing, consumption of fluids 2 h prior to surgery.

### Supplementary Information

**Additional file 1: Appendix A.** Patient Satisfaction Survey.

## Data Availability

The dataset is potentially available upon request.
