# Supplementary material for: Randomized controlled trial of liberal vs. standard fasting instructions in percutaneous cardiac procedures
Source: Perioper Med (Lond). 2023 Aug 8;12:44. doi: 10.1186/s13741-023-00333-z (PMC10408039; doi:10.1186/s13741-023-00333-z)
Supplement: Supplementary file 1 — Additional file 1: Appendix A. Patient Satisfaction Survey. [file 13741_2023_333_MOESM1_ESM.docx]

**Appendix A: Patient Satisfaction Survey**

1. When was the last time you ate? __________________________________________
2. When was the last time you drank something? _______________________________
3. What was the last thing you drank? ________________________________________
4. When was the last time you took oral medication with water? ___________________
5. Do you normally drink caffeinated beverages? Yes / No (Circle)

Please rate the following by choosing the number between 0 and10 that best describes you, where **0 indicates the least and 10 indicates the most**.

1. **How thirsty are you right now?**

Not Extreme

At all ☺ Thirst ☹

0 1 2 3 4 5 6 7 8 9 10

1. **How hungry are you right now?**

Not Extreme

At all ☺ Hunger ☹

_____________________________________________________________________

0 1 2 3 4 5 6 7 8 9 10

1. **Do you currently have a headache?**

Not Extreme

At all ☺ headache☹

_____________________________________________________________________

0 1 2 3 4 5 6 7 8 9 10

1. **What is the most nauseated you have felt today?**

Not Extreme

At all ☺ Nausea ☹

0 1 2 3 4 5 6 7 8 9 10

1. **How lightheaded do you feel today?**

Not Very

At all ☺ Light-headed ☹

_____________________________________________________________________

0 1 2 3 4 5 6 7 8 9 10

1. **How anxious do you feel today?**

Not Very

At all ☺ Anxious ☹

_____________________________________________________________________

0 1 2 3 4 5 6 7 8 9 10
